# Efficacy of Selected Bacterial Strains in the Protection and Growth Stimulation of Winter Wheat and Maize

**DOI:** 10.3390/plants14050636

**Published:** 2025-02-20

**Authors:** Arkadiusz Filipczak, Łukasz Sobiech, Agnieszka Wita, Roman Marecik, Wojciech Białas, Agnieszka Drożdżyńska, Monika Grzanka, Jakub Danielewicz, Piotr Szulc

**Affiliations:** 1 Department of Agronomy, Faculty of Agriculture, Horticulture and Biotechnology, Poznań University of Life Sciences, Dojazd 11, 60-632 Poznan, Poland; arkadiusz.filipczak@up.poznan.pl (A.F.); monika.grzanka@up.poznan.pl (M.G.); piotr.szulc@up.poznan.pl (P.S.); 2 Department of Biotechnology and Food Microbiology, Poznan University of Life Sciences, Wojska Polskiego 48, 60-627 Poznan, Poland; agnieszka.wita@up.poznan.pl (A.W.); roman.marecik@up.poznan.pl (R.M.); wojciech.bialas@up.poznan.pl (W.B.); agnieszka.drozdzynska@up.poznan.pl (A.D.); 3 Department of Mycology, Institute of Plant Protection—National Research Institute, Władysława Wegorka 20, 60-318 Poznan, Poland; j.danielewicz@iorpib.poznan.pl

**Keywords:** biofungicide, biostimulant, seed dressing, *Fusarium* control, *Bacillus* sp., *Paenibacillus* sp., *Pseudomonas* sp., plant chlorophyll fluorescence

## Abstract

The use of biopreparations currently plays a significant role in limiting the use of plant protection products and fertilizers. In this study, preparations based on *Bacillus velezensis_KT27*, *Paenibacillus polymyxa*, *Pseudomonas synxatha,* and a mixture of *Bacillus subtilis*, *Pseudomonas simiae*, and *Bacillus velezensis_S103*, used as seed dressings at doses of 0.5 L and 1.0 L × 100 kg^−1^ grain, were tested to determine their efficacy. The prothioconazole preparation was used for comparison as a synthetic fungicide. The test microorganisms were prepared as standardized preparations diluted with sterile water to obtain a final cell concentration of 5 × 10^8^ CFU/mL for each bacterial strain. The ability of selected bacterial strains to solubilize phosphate was quantitatively evaluated as one of the factors influencing the stimulation of crop growth. The obtained results indicate that the microorganisms can reduce the infection in seedlings, and the health of those seedlings depends on the preparation used and its dose. The tested microorganisms had a positive effect on plant growth, which was confirmed by the analyses of plant height, fresh mass, and chlorophyll fluorescence. The results indicate that the selected strains of microorganisms *Bacillus* ssp., *Paenibacillus* ssp., and *Pseudomonas* ssp. may be used in the protection and growth stimulation of crop plants, but this needs to be verified by field tests prior to their practical application.

## 1. Introduction

Global grain production is responsible for maintaining global food security [1]. Wheat plays a very important role in providing food for humans and animals as a source of protein and calories [2]. Maize, in addition to its global economic importance, has been a model organism in many studies over the years. Biotic stresses severely limit yields of both wheat and maize [3,4]. Under natural field conditions, crop plants are often exposed to a combination of factors causing stress and pathogen infestation [5]. Pathogenic microorganisms and the diseases they cause destabilize and limit the quantitative and qualitative production of cereals [6,7].

Some of the most dangerous pathogens are fungi of the genus *Fusarium* due to their infestation rate of crop plants and their high contamination of cereal grains with mycotoxins. One of the plants most infected by pathogens is winter wheat [2]. Many *Fusarium* species are responsible for the formation of mycotoxins, and they can infect roots, stems, leaves, and ears at various growth stages [8]. *Fusarium graminearum* and *Fusarium culmorum* are species of fungi responsible for the production of trichothecene mycotoxins, including nivalenol (NIV) and deoxynivalenol (DON) [9]. *Fusarium avenaceum* is also frequently found in cereal plantations, e.g., wheat, oats, and barley, producing mycotoxins such as moniliformins, chrysogins, aurofusarin, and acuminatopyron [10,11]. *Fusarium fujikuroi* is a pathogen that synthesizes significant amounts of gibberellins as well as harmful compounds such as fusaric acid, fusarins, bicaverins, and fusarubins [12].

There are many commercially available fungicides intended for seed treatment. Consequently, seed dressing is one of the methods applied to combat fungal diseases transmitted through seeds and soil. However, due to the toxic effects of these agents, seed treatments may also negatively affect the vigor of crop plants [13]. Active substances exhibiting fungicide activity also include prothioconazole, which belongs to the triazole group [14]. The main effect of triazoles is to inhibit the 14-alpha-demethylation of lanosterol in the pathway of ergosterol biosynthesis [15]. Ergosterol is the most important sterol building block of fungal membranes, and it is necessary for their development [16]. The common use of phytopathogen control by chemical methods results in growing concerns about environmental pollution and adverse side effects on human and animal health. It also contributes to the development of pathogen resistance [17,18,19].

Biopesticides are substances of plant and animal origin, microorganisms, and their derivatives. These substances have lost their importance due to the high effectiveness and quick effect of chemical protection products. However, biopesticides are characterized by low toxicity to organisms, including humans, and cause no resulting environmental pollution [20]. For this reason, the use of beneficial microorganisms has become an alternative approach in biological plant protection [19]. A number of studies are being carried out on the applicability of biopesticides in plant biostimulation and protection, and the obtained results are promising. Increasing attention is also focused on the benefits of using mixtures of chemical plant protection products with biopesticides [21]. The effectiveness of biopesticides can be improved using different mixtures of microorganisms or their different formulations, as well as improving their physiology and genetics [19]. In terms of efficacy, the appropriate selection of beneficial microorganisms is also important in terms of their ability to be used under specific soil, humidity, and thermal conditions [22].

The bacterium *Bacillus velezensis* belongs to the plant-growth-promoting rhizobacteria; it stimulates the growth of crop plants and provides biocontrol. Such actions are possible because these bacteria secrete secondary metabolites such as lipopeptides and polyketides. These compounds not only inhibit the development of pathogens but can also stimulate induced systemic immunity [23]. *Paenibacillus polymyxa* is classified as a potential plant-growth-promoting rhizobacterium [24]. *Paenibacillus polymyxa*, by producing phytohormones and exopolysaccharides, increases nutrient availability and supports and stimulates plant growth [25]. *Pseudomonas* rhizobacteria stimulate plant development and biocontrol directly or indirectly through the production of protective enzymes and antibiotics. *Pseudomonas synxantha* is responsible for the synthesis of volatile organic compounds and phenazine-1-carboxylic acid, exhibiting fungicidal properties [26]. *Bacillus subtilis* is a microorganism producing ammonia, indoleacetic acid, and siderophores. It is capable of solubilizing phosphorus and producing hydrogen cyanide, thus supporting plant growth [27]. *Pseudomonas simiae* produces siderophores that chelate iron from the environment, enhancing its availability to plants while simultaneously restricting access to this element [28].

Crops are exposed to adverse environmental conditions. One of these is the presence of pathogens, which can significantly reduce the quality and quantity of crop yields [5,9,29]. A plant’s response to environmental stresses can be determined based on the measurement of chlorophyll fluorescence [30]. This method provides non-invasive measurements and is characterized by high sensitivity and reliability of the results. This method is often selected to measure the intensity of the impact of stresses on plants, such as those caused by pathogens, and the phytotoxic effects of plant protection products, including mortars [5,29,31,32]. The light energy absorbed by chlorophyll molecules is only partially used for the photosynthesis reaction. The remaining energy is lost as heat or emitted as chlorophyll fluorescence. These processes are closely interconnected, with changes in the photosynthesis process easily detected by measuring chlorophyll fluorescence [33].

The aim of the study was to evaluate the effects of various isolated microorganisms compared to a synthetic fungicide on seed germination, *Fusarium* pathogen control, plant chlorophyll fluorescence, plant vigor, growth stimulation, and fresh weight of winter wheat and maize.

## 2. Results

### 2.1. Phosphate-Dissolving Activity and Fungistatic Activity of Selected Bacterial Strains

A total of 212 culturable rhizosphere and endophytic bacterial strains were isolated from samples collected both from agricultural areas in the Wielkopolska province, Poland, between 2002 and 2020, and from compost samples. These isolates were functionally screened to assess their ability to convert insoluble soil phosphorus into its bioavailable forms and their antifungal activity. Based on preliminary in vitro screening results, six pure bacterial cultures were selected for further analyses. Detailed results of the screening tests for the six selected strains with the most promising properties are presented below. The strains chosen for detailed investigations included *Bacillus velezensis KT27*, *Paenibacillus polymyxa*, *Pseudomonas synxantha*, *Bacillus subtilis*, *Pseudomonas simiae*, and *Bacillus velezensis S103*.

**Figure 1:**
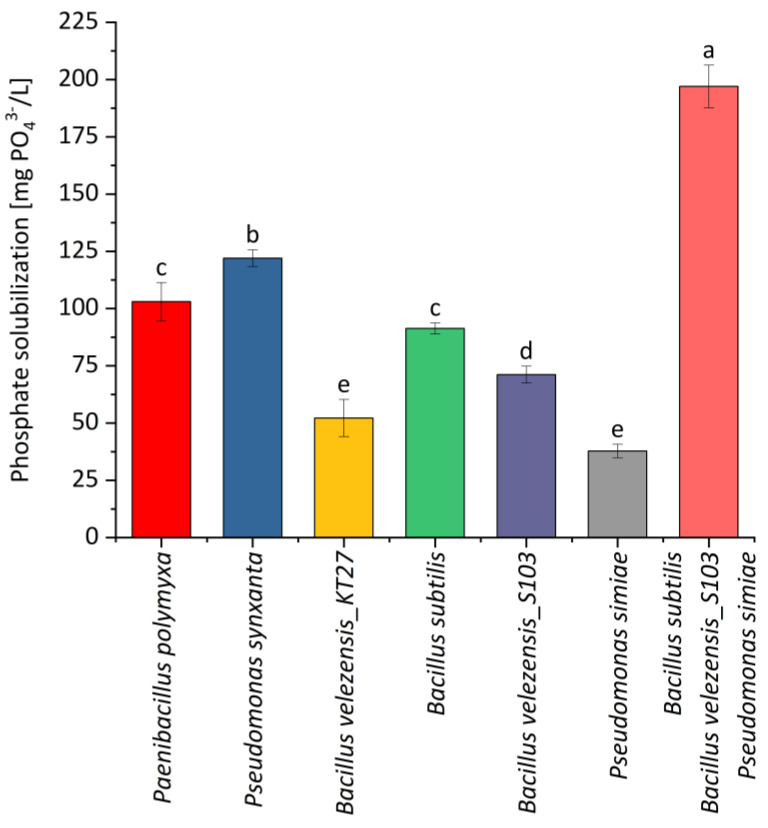
Effects of various microbial preparations on available phosphorus after six days of incubation at 30 °C. Different letters indicate statistically significant differences in mean values based on the LSD test (*p* < 0.05).

Among the individual strains, *Pseudomonas synxantha* exhibited high phosphate solubilization activity, ranking second only to the combination treatments and showing statistically significant differences compared to other strains (Figure 1). Similarly, *Bacillus subtilis* and *Paenibacillus polymyxa* displayed relatively high phosphate solubilization levels. In contrast, *Bacillus velezensis_KT27* and *Pseudomonas simiae* demonstrated the lowest phosphate solubilization capacities, performing significantly less effectively than all the other treatments. This highlights their limited potential for phosphate solubilization under the adopted experimental conditions. The combination of *Bacillus subtilis*, *Bacillus velezensis_S103*, and *Pseudomonas simiae* exhibited the highest phosphate solubilization levels, significantly exceeding the performance of all the individual strains and other combinations. This observation suggests that interactions among these microbial species synergistically enhance phosphate solubilization activity. Notably, the inclusion of *Bacillus velezensis_S103* in the most effective combination likely contributed to enhanced solubilization, potentially due to its strong metabolic activity or synergistic interactions with the other species. This highlights the potential for specific strain combinations to outperform individual strains in phosphate solubilization.

The results presented in Table 1 indicate that *Bacillus velezensis KT27* and *Paenibacillus polymyxa* exhibited the most potent antifungal activity, particularly against *Fusarium graminearum* (50 mm inhibition zone) and *Fusarium fujikuroi* (50 mm for *P. polymyxa*). Other strains, such as *Pseudomonas synxantha*, *Bacillus subtilis*, and *Pseudomonas simiae*, demonstrated moderate inhibition capacity against *Fusarium fujikuroi* and *Fusarium avenaceum*, with inhibition zones ranging from 14 mm to 20 mm. Notably, *Fusarium culmorum* appeared to be the least affected, as only *Bacillus subtilis* and *Pseudomonas simiae* exhibited any inhibitory effects (12 mm clear zones). The evaluation of the cell-free supernatant after centrifugation and filtration revealed that the antifungal activity was generally lower in the absence of bacterial cells. However, *Bacillus velezensis* retained significant inhibitory effects against *Fusarium graminearum* (50 mm) and moderate effects against *Fusarium fujikuroi* (24 mm) and *Fusarium avenaceum* (14 mm). Similarly, *Paenibacillus polymyxa* showed a slightly reduced, but notable activity against *Fusarium graminearum* (40 mm) and *Fusarium fujikuroi* (40 mm). The inhibitory effects of *Pseudomonas synxantha*, *Bacillus subtilis*, and *Pseudomonas simiae* were also observed in the supernatant fraction, albeit at lower levels compared to the culture fluid. The results suggest that a direct bacterial interaction or the presence of bacterial cells enhances antifungal activity, likely due to the secretion of bioactive compounds. The retention of the activity in the cell-free supernatant for certain strains indicates that diffusible antifungal metabolites contribute to fungal growth suppression. These findings highlight the importance of *Bacillus velezensis*, *Paenibacillus polymyxa*, and *Bacillus subtilis* as promising candidates for biocontrol applications against *Fusarium* species in agricultural settings.

### 2.2. Seed Germination and Health Parameters of Plant Seedlings (Seeds Inoculated with Fusarium)

**Figure 2:**
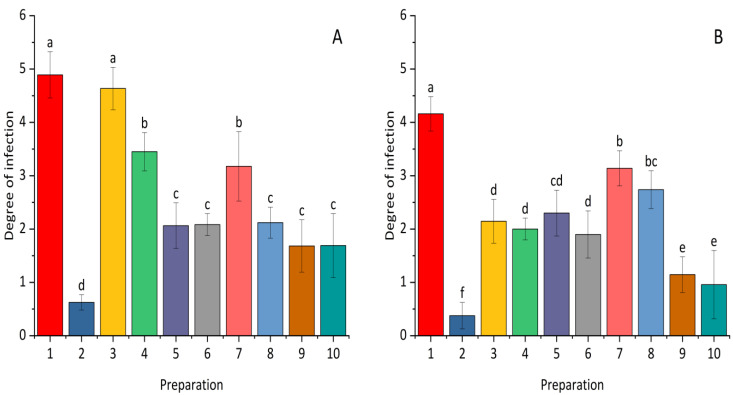
The effect of *Bacillus* ssp., *Paenibacillus* ssp., *Pseudomonas* ssp., or a synthetic fungicide on the degree of infection with Fusarium pathogens in maize (**A**) and winter wheat (**B**). 1—control; 2—(0.1 L) prothioconazole; 3 (0.5 L), 4 (1.0 L)—*Bacillus velezensis_KT27*; 5 (0.5 L), 6 (1.0 L)—*Paenibacillus polymyxa*; 7 (0.5 L), 8 (1.0 L)—*Pseudomonas synxatha*; 9 (0.5 L), 10 (1.0 L)—*Bacillus subtilis*, *Pseudomonas simiae*, *Bacillus velezensis_S103*. The doses of the preparations in combinations 1–10 are consistent with the numbers and values given in Section 4. Different letters indicate statistically significant differences in mean values based on the LSD test (*p* < 0.05).

Statistical analysis of the conducted experiments showed significant differences in the use of preparations containing selected bacterial strains and a synthetic fungicide in the level of infection of the tested plants (Figure 2). Prothioconazole showed the highest level of efficacy in reducing the infection of winter wheat and maize. The tested plants treated with selected bacterial strains had different levels of infection with *Fusarium* strains depending on the type of plant, the preparation used, and the dose. In the case of maize, the most effective microorganisms were *Paenibacillus polymyxa*, a higher dose of *Pseudomonas synxatha*, and a mixture of *Bacillus subtilis*, *Pseudomonas simiae*, and *Bacillus velezensis_S103*. The infection of winter wheat seedlings was limited non-chemically to the greatest extent by the mixture of *Bacillus subtilis*, *Pseudomonas simiae*, and *Bacillus velezensis_S103*.

The results recorded in the germination experiment indicate that the preparations used did not have a statistically significant effect on the germination capacity of winter wheat grains inoculated with Fusarium (Table 2). A statistically different, and the lowest result for the germination energy of winter wheat grains inoculated with Fusarium was obtained only for the Bacillus subtilis, Pseudomonas simiae, Bacillus velezensis_S103 mixture applied at the dose of 1.0 L × 100 kg^−1^ of grain. Statistical analysis of the results showed no significant differences between the use of preparations containing selected bacterial strains and the synthetic fungicide on the germination energy and germination capacity of maize grain inoculated with Fusarium.

**Figure 3:**
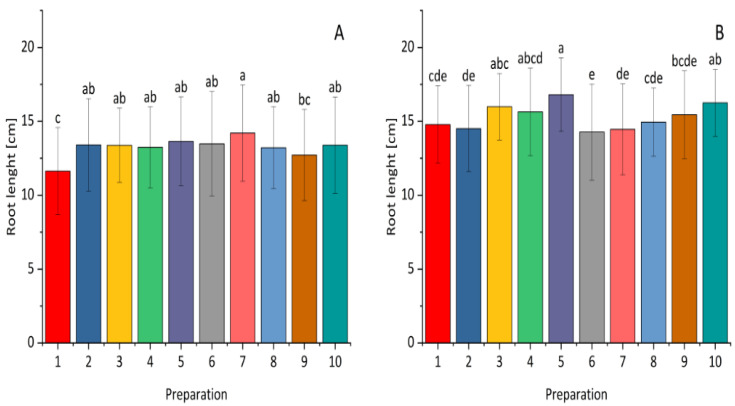
The effect of *Bacillus* ssp., *Paenibacillus* ssp., *Pseudomonas* ssp., or the synthetic fungicide on root length in maize (**A**) and winter wheat (**B**) seeds inoculated with *Fusarium*. 1—control; 2—(0.1 L) prothioconazole; 3 (0.5 L), 4 (1.0 L)—*Bacillus velezensis_KT27*; 5 (0.5 L), 6 (1.0 L)—*Paenibacillus polymyxa*; 7 (0.5 L), 8 (1.0 L)—*Pseudomonas synxatha*; 9 (0.5 L), 10 (1.0 L)—*Bacillus subtilis*, *Pseudomonas simiae*, *Bacillus velezensis_S103*. The doses of the preparations in combinations 1–10 are consistent with the numbers and values given in Section 4. Different letters indicate statistically significant differences in mean values based on the LSD test (*p* < 0.05).

The used preparations exhibited varying levels of their effect on the length of plant roots. Application of all the preparations except for combination 9—*Bacillus subtilis*, *Pseudomonas simiae*, *Bacillus velezensis_S103* at a dose of 0.5 L resulted in better maize root growth compared to the control (Figure 3). In the case of winter wheat, the greatest seedling root lengths were obtained after the application of a lower dose of *Paenibacillus polymyxa* and a higher dose of the mixture of *Bacillus subtilis*, *Pseudomonas simiae*, and *Bacillus velezensis_S103*.

**Figure 4:**
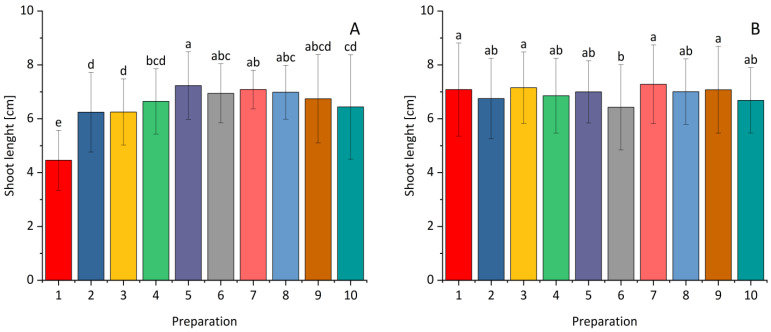
The effect of *Bacillus* ssp., *Paenibacillus* ssp., *Pseudomonas* ssp., or the synthetic fungicide on shoot length of maize (**A**) and winter wheat (**B**) seeds inoculated with *Fusarium*. 1—control; 2—(0.1 L) prothioconazole; 3 (0.5 L), 4 (1.0 L)—*Bacillus velezensis_KT27*; 5 (0.5 L), 6 (1.0 L)—*Paenibacillus polymyxa*; 7 (0.5 L), 8 (1.0 L)—*Pseudomonas synxatha*; 9 (0.5 L), 10 (1.0 L)—*Bacillus subtilis*, *Pseudomonas simiae*, *Bacillus velezensis_S103*. The doses of the preparations in combinations 1–10 are consistent with the numbers and values given in Section 4. Different letters indicate statistically significant differences in mean values based on the LSD test (*p* < 0.05).

In the case of the shoot length of the inoculated maize grains, the results showed statistical differences in the treatment combinations compared to the control (Figure 4). All the bacterial strains showed statistical differences in the tested parameter compared to the control for maize. The highest value of this parameter for maize was recorded after applying *Paenibacillus polymyxa* at a dose of 0.5 L × 100 kg^−1^ grain. There were no statistical differences in shoot length for winter wheat between the control and individual selected bacterial strains, with the exception of *Paenibacillus polymyxa* at a dose of 1.0 L × 100 kg^−1^ grain where the value of the tested parameter was the lowest.

**Figure 5:**
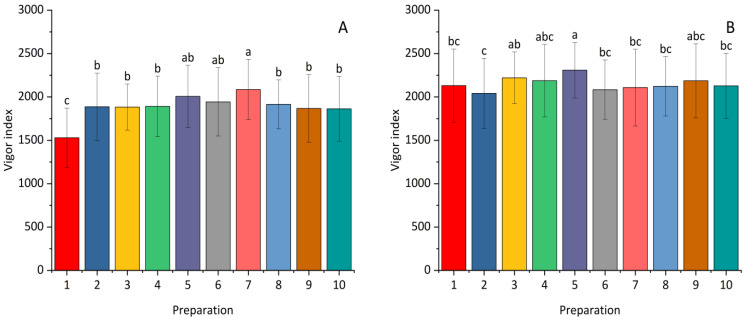
The influence of *Bacillus* ssp., *Paenibacillus* ssp., *Pseudomonas* ssp., or the synthetic fungicide on the vigor index of maize (**A**) and winter wheat (**B**) seeds inoculated with *Fusarium*. 1—control; 2—(0.1 L) prothioconazole; 3 (0.5 L), 4 (1.0 L)—*Bacillus velezensis_KT27*; 5 (0.5 L), 6 (1.0 L)—*Paenibacillus polymyxa*; 7 (0.5 L), 8 (1.0 L)—*Pseudomonas synxatha*; 9 (0.5 L), 10 (1.0 L)—*Bacillus subtilis*, *Pseudomonas simiae*, *Bacillus velezensis_S103*. The doses of the preparations in combinations 1–10 are consistent with the numbers and values given in Section 4. Different letters indicate statistically significant differences in mean values based on the LSD test (*p* < 0.05).

The results showed a statistically significant effect of selected preparations on the vigor index of winter wheat and maize (Figure 5). All the applied preparations positively affected the tested parameter in maize in comparison with the control treatment. The highest values of the maize vigor index were recorded after the use of *Paenibacillus polymyxa* and a lower dose of *Pseudomonas synxatha.* The best plant vigor effect for winter wheat, statistically different from that in the control, was obtained after seed treatment with *Paenibacillus polymyxa* at a lower dose.

### 2.3. The Effect of Selected Bacterial Strains on Plant Growth Under Greenhouse Conditions

The experiment showed a statistically significant effect of selected preparations on the height of winter wheat and maize plants (Table 3). The results depended on the crop plant, the dose of the preparation, and the measurement time. The highest values of winter wheat height were recorded for the combination where *Paenibacillus polymyxa* at a dose of 0.5 L and *Pseudomonas synxatha* at a dose of 1.0 L × 100 kg^−1^ grain were used in the first measurement. In the second measurement, the greatest value of this parameter was obtained after the application of *Bacillus velezensis_KT27* at a dose of 0.5 L × 100 kg^−1^ grain, while it was the lowest for *Pseudomonas synxatha* at a dose of 0.5 L × 100 kg^−1^ grain. The combination using *Pseudomonas synxatha* at a dose of 0.5 L × 100 kg^−1^ grain provided the highest maize height, which was statistically different from the control at 18 DAS and 28 DAS. The same result was observed for *Bacillus velezensis_KT27* at a dose of 0.5 L × 100 kg^−1^ grain at 28 DAS.

**Figure 6:**
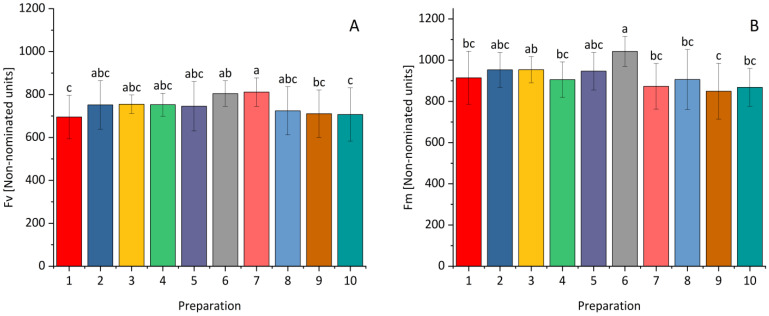
The influence of *Bacillus* ssp., *Paenibacillus* ssp., *Pseudomonas* ssp., or the synthetic fungicide on Fv—variable fluorescence (**A**) and Fm—maximum fluorescence (**B**) of winter wheat. 1—control; 2—(0.1 L) prothioconazole; 3 (0.5 L), 4 (1.0 L)—*Bacillus velezensis_KT27*; 5 (0.5 L), 6 (1.0 L)—*Paenibacillus polymyxa*; 7 (0.5 L), 8 (1.0 L)—*Pseudomonas synxatha*; 9 (0.5 L), 10 (1.0 L)—*Bacillus subtilis*, *Pseudomonas simiae*, *Bacillus velezensis_S103*. The doses of the preparations in combinations 1–10 are consistent with the numbers and values given in Section 4. Different letters indicate statistically significant differences in mean values based on the LSD test (*p* < 0.05).

The applied preparations exhibited varying effects on the variable and maximum fluorescence of winter wheat (Figure 6). A statistically significant increase in Fv compared to the control was noted after the application of a higher dose of *Paenibacillus polymyxa* and a lower dose of *Pseudomonas synxatha*, and for the Fm parameter after dressing the grain with a higher dose of *Paenibacillus polymyxa*.

**Figure 7:**
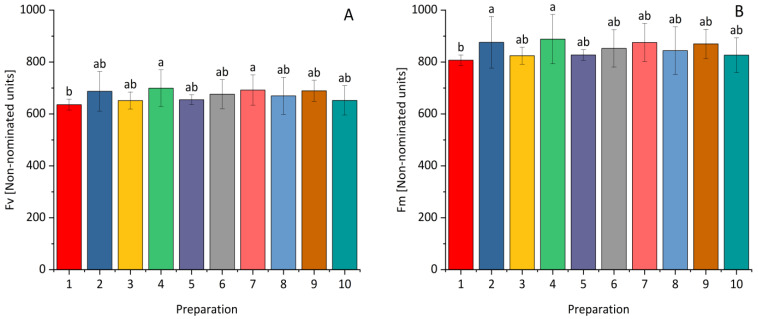
The influence of *Bacillus* ssp., *Paenibacillus* ssp., *Pseudomonas* ssp., or the synthetic fungicide on Fv—variable fluorescence (**A**) and Fm—maximum fluorescence (**B**) of maize. 1—control; 2—(0.1 L) prothioconazole; 3 (0.5 L), 4 (1.0 L)—*Bacillus velezensis_KT27*; 5 (0.5 L), 6 (1.0 L)—*Paenibacillus polymyxa; 7* (0.5 L), *8* (1.0 L)—*Pseudomonas synxatha*; 9 (0.5 L), 10 (1.0 L)—*Bacillus subtilis*, *Pseudomonas simiae*, *Bacillus velezensis_S103*. The doses of the preparations in combinations 1–10 are consistent with the numbers and values given in Section 4. Different letters indicate statistically significant differences in mean values based on the LSD test (*p* < 0.05).

For maize, a statistically significant increase in Fv compared to the control was recorded after the application of a higher dose of *Bacillus velezensis_KT27* and a lower dose of *Pseudomonas synxatha*, while for the Fm parameter, it was after dressing the grain with prothioconazole and a higher dose of *Bacillus velezensis_KT27*. (See Figure 7).

The results showed a statistically significant effect of selected preparations on the maximum photochemical efficiency of photosystem II in winter wheat (Table 4). The highest value of this parameter for winter wheat was found after the application of *Bacillus velezensis_KT27* and *Paenibacillus polymyxa* at a dose of 1.0 L × 100 kg^−1^ grain, *Pseudomonas synxatha* and the preparation from *Bacillus subtilis*, *Pseudomonas simiae*, and *Bacillus velezensis_S103* at doses of 0.5 L and 1.0 L × 100 kg^−1^ grain. Statistical analysis of the results showed no significant differences in the use of preparations containing *Bacillus* spp., *Paenibacillus* spp., *Pseudomonas* spp., or the synthetic fungicide on maximum photochemical efficiency of photosystem II in maize.

Statistical analysis showed significant differences in the fresh weight of maize and winter wheat treated with selected bacterial strains. The results depended on the tested crop, the strains used, and their dosage. The greatest fresh weight of winter wheat plants was recorded after the application of *Bacillus velezensis_KT27* and *Paenibacillus polymyxa* in two doses (Table 5). All the microbiological preparations increased the fresh weight of maize plants, which is confirmed by the results. Statistical differences in this parameter were found between all the combinations except for prothioconazole at a dose of 0.1 L and the *Bacillus subtilis*, *Pseudomonas simiae*, *Bacillus velezensis_S103* mixture at a dose of 1.0 L × 100 kg^−1^ grain for maize compared to the control.

## 3. Discussion

*Fusarium* pathogens negatively affect the quantity and quality of crops. They reduce cereal yields and, in addition, contaminate grain with mycotoxins [34]. One method to limit the development of pathogens involves seed treatment. Synthetic plant protection products are often used for this purpose [35].

In the selection of bacterial strains intended for the production of biopreparations that promote plant growth and suppress pathogen development without the need for chemical plant protection agents, it is essential to consider specific metabolic activities. These include the production of phytohormones (e.g., auxins), solubilization of insoluble forms of essential nutrients such as phosphorus, and antifungal activity [36,37]. Selected strains need to demonstrate the ability to colonize the root system, withstand adverse environmental conditions, and be compatible with the soil microbiome. Studies indicate that bacteria exhibiting antifungal activity, e.g., by producing antibiotic metabolites or enzymes that degrade fungal cell walls, often also possess the ability to solubilize phosphorus. These traits are characteristic of such bacterial genera as *Pseudomonas*, *Bacillus*, *Peanibacillus*, and *Streptomyces*, which are frequently isolated from the rhizosphere of cultivated plants [38,39]. Scientists have noticed, however, that individual regions of the world are characterized by different microbial species compositions [40]. Their action varies depending on the specific strain of microorganisms [41]. The study compared the effect of the application of various preparations containing *Bacillus* spp., *Paenibacillus* spp., *Pseudomonas* spp., and synthetic triazole on the growth and development of winter wheat and maize.

In the study, prothioconazole limited the development of *Fusarium* pathogens to the greatest extent, which is consistent with other literature sources [42,43], where triazole was reported to have fungistatic properties. Prothioconazole is also commonly used as a seed treatment against *Fusarium* in various crops [44]. The widespread application of chemicals leads to the development of resistant pathogen populations [45] and pollution of various ecosystems. Chemicals are toxic to various organisms, including humans. An important element of global food production is caring for the environment. Maintaining the quality of products and protecting the biodiversity of ecosystems require alternative means of production, which are available and may become urgently needed [46]. The effectiveness of microbial preparations turned out to be lower than that of synthetic fungicides. This may be because microorganisms are sensitive to various environmental conditions [47,48]. Chen et al. [49] showed that *Bacillus velezensis* has an antagonistic effect against *F. graminearum* in wheat, which is confirmed by our research, and also against *F. oxysporum* [50]. In turn, *Paenibacillus polymyxa* containing the biofilm polysaccharide D-glucuronate is an antagonist for *F. graminearum* [51], which is in agreement with the results presented in this study. The preparation containing *Bacillus subtilis*, *Pseudomonas simiae*, and *Bacillus velezensis_S103* exhibited the highest *Fusarium* inhibitory activity in winter wheat compared to the other biocontrols. *Bacillus subtilis*, by producing siderophores, proteases, catalase, and chitinase, showed an antagonistic effect against *F. proliferatum*, *F. culmorum*, *F. verticillioides*, and *F. oxysporum* [52]. The fengycin produced by these microorganisms destroys *F. graminearum* [53]. The development of *Fusarium graminearum* is strongly inhibited by *Bacillus velezensis_S103*, which produces lipopeptides: bacilomycin D, iturin, and plipastatin, which exhibit fungistatic properties [54,55]. The preparations containing microorganisms had no negative effect on the germination energy and capacity of winter wheat and maize grains. Only the mixture of *Bacillus subtilis*, *Pseudomonas simiae*, and *Bacillus velezensis_S103* at a dose of 1 l × 100 kg^−1^ grain statistically significantly reduced the germination energy of winter wheat seeds. Nevertheless, the selected bacterial strains did not statistically reduce the germination capacity of the seeds. This result is confirmed by other studies [56,57], where the authors demonstrated a varying effect on energy and germination capacity depending on the microorganisms used but without a negative impact on the outcome.

The experiment showed a positive effect of *Bacillus velezensis_KT27* at a dose of 0.5 L on the height of maize plants. The fresh weight of plants increased after applying two doses of *Bacillus velezensis_KT27* in winter wheat. In turn, *Bacillus velezensis_KT27* at a dose of 1.0 L × 100 kg^−1^ grain enhanced maximum photochemical efficiency in winter wheat, thus improving the activity of the photosynthetic apparatus. These findings are consistent with previous studies, which documented similar growth-promoting effects of *Bacillus velezensis*. For instance, its application has been shown to increase the fresh weight of radish roots and leaves [58]. The observed biomass enhancement has been attributed to *Bacillus* activity at the level of 1-aminocyclopropane-1-carboxylate (ACC) deaminase, as well as the production of indole-3-acetic acid (IAA) and ammonia, mechanisms previously reported in the literature. Furthermore, *Bacillus velezensis* has been associated with increased root and shoot biomass in cucumbers [59]. Studies have demonstrated an increase in phosphorus content in maize shoots, which has been linked to the presence of phosphatase genes in *Bacillus velezensis*, facilitating phosphate solubilization and improving nutrient uptake efficiency [60]. In addition to its growth-promoting properties, *Bacillus velezensis* produces siderophores, chitinase, and protease, compounds known for their role in plant protection. Research has shown its antifungal activity against *Fusarium* pathogens, highlighting its potential in agricultural applications [61]. The consistency of these findings across multiple studies underscores the significance of *Bacillus velezensis* as a beneficial microbial agent.

In this study, the use of *Paenibacillus polymyxa* at both doses had a positive effect on the plant height, vigor index, and fresh weight of maize. Importantly, the use of selected bacterial strains promoted the shoot and root length in maize plants to varying degrees. The experiment showed a positive effect of *Paenibacillus polymyxa* at a dose of 0.5 L × 100 kg^−1^ grain on root length, vigor index, and fresh weight in winter wheat. In turn, *Paenibacillus polymyxa* at a dose of 1.0 L × 100 kg^−1^ grain increased the maximum photochemical efficiency of photosystem II and the fresh weight of winter wheat. This is confirmed by another study, where only some of the *Paenibacillus polymyxa* strains resulted in significant differences compared to the control in terms of plant height, fresh root weight, and total weight of pepper plants [41]. The differences between strains of microorganisms should be emphasized in the case of the production activity of indole-3-acetic acid, and the role of genotype formation (only some strains have a nitrogen-fixing gene), which results in different effects in promoting plant growth. The authors suggest that *Paenibacillus polymyxa* synthesizes other growth factors. Padda et al. [62] showed that canola seeds inoculated with *Paenibacillus polymyxa* significantly differed from the control in terms of the length and dry weight of their roots and shoots. Additionally, they reported that plants treated with these microorganisms were characterized by a higher nitrogen content in their leaves.

In this experiment the influence of *Pseudomonas synxantha* on the growth and development of plants was varied. This is primarily due to the type of crop plant. Maize plants grown from seeds treated with *Pseudomonas synxantha* were taller and showed greater fresh weight, vigor index, and length of roots and shoots. Such results were not confirmed in the case of winter wheat, where the recorded values depended on the tested trait and preparation dose. Similar results were reported by [63], where the use of *Pseudomonas* ssp. influenced the growth of individual crop plants in different soil environments to varying degrees. The authors of that study emphasized that the obtained differences in growth results were related to the different sensitivity of crop plants to individual microorganisms. In their experiments, Ganesh et al. [64] found a significant impact of *Pseudomonas* on the shoot biomass of *Arabidopsis thaliana*. According to their findings, individual *Pseudomonas* strains were able to fix nitrogen and produce indolyl 3-acetic acid, ammonia, and catalase to varying degrees. They showed different ACC deaminase and protease activities, which may indicate a varying impact of *Pseudomonas* strains on promoting plant growth.

The use of the *Bacillus subtilis*, *Pseudomonas simiae*, *and Bacillus velezensis_S103* mixture had various effects on the tested plants. A dose of 0.5 L increased shoot length and the vigor index in maize, the maximum photochemical efficiency of photosystem II in winter wheat, and fresh weight in maize. Improving the activity of the photosynthetic apparatus may indicate a better condition of the plant. The 1.0 L dose affected only the root length, the shoot length, and the vigor index in maize. Some studies have confirmed a positive effect of *Bacillus subtilis* on the growth of cucumber seedlings and the photosynthesis system [65], *Sesamum indicum* L. [27], and a positive effect of *Bacillus velezensis_S103* on the growth of *Arabidopsis thaliana* [66]. However, there are also some scientific works where *Pseudomonas simiae* did not promote wheat root development [67] or the development of *Miscanthus × giganteus* shoots [68].

The experiments conducted in this study demonstrated that microbial combinations can significantly enhance phosphate solubilization compared to their individual strains. While *Pseudomonas synxantha* and *Paenibacillus polymyxa* showed considerable potential as individual agents, the combined effects of mixed strains, particularly those involving *Bacillus subtilis*, *Bacillus velezensis_S103*, and *Pseudomonas simiae*, emphasize the importance of microbial synergy in optimizing phosphorus bioavailability. Phosphorus plays an important role in the process of photosynthesis. It is a component of the structure of nucleic acids and adenosine phosphates. Phosphorus is an essential macronutrient in the transport of energy and carbohydrates [69]. Research has demonstrated that microbial consortia can significantly enhance phosphate solubilization compared to individual strains. For instance, a study formulated a microbial consortium consisting of a straw-degrading bacterium and a phosphate-solubilizing bacterium, which exhibited improved straw degradation and phosphorus solubilization [70]. Another investigation highlighted that the application of phosphate-solubilizing bacteria (PSB) consortia promoted maize growth more effectively than single-strain inoculations, indicating the superior efficacy of microbial combinations [71]. These findings underscore the potential of utilizing microbial consortia to optimize phosphate solubilization and improve plant growth.

As predicted by Dent [72], the share of biological preparations in the plant protection products market will increase in the coming years. This gives us a meaningful opportunity to protect the natural environment.

## 4. Materials and Methods

### 4.1. Sample Collection, Bacteria Isolation, Identification, and Selection

Plant samples were collected from agricultural areas in the Wielkopolska province, Poland, between 2002 and 2020 to isolate culturable rhizosphere and endophytic bacteria. Microorganisms were isolated from habitats where the following plants were grown: beetroot, white mustard, field pea, blueberry, cabbage, corn, lupine, rapeseed, oil radish, soybean, and wheat. Additionally, isolation was carried out from compost obtained through the fermentation and composting process of selectively collected biodegradable waste, such as green landscaping waste from the maintenance of green areas, gardens, and parks, as well as kitchen waste.

Endophytic bacteria were isolated from stems, roots, and symbiotic root nodules of plants. The stems were cut into 2 cm fragments, while the roots were cleaned of adhering soil, rinsed three times with sterile distilled water, and then the symbiotic root nodules were separated using a sterile blade. Two distinct surface sterilization protocols were employed to prepare samples for bacterial isolation. According to procedure I, the tissue fragments were shaken for 15 min in a 1% Tween 80 solution. They were then transferred successively to a solution of sodium hypochlorite (0.01%), H_2_O_2_ (10%), and ethanol (70%). Each tissue immersion lasted 2 min. Afterward, 2 cm fragments of stems, roots, and symbiotic root nodules were rinsed three times in sterile distilled water. According to procedure II, 2 cm pieces of plant tissue were immersed in 95% ethanol for 10 s and 3% sodium hypochlorite for 4 min, respectively, and then rinsed five times in sterile distilled water. After the surface sterilization, the plant tissues were transferred with sterile forceps to mortars containing 9 mL of physiological saline, and they were ground. The obtained homogenate was the starting material for the isolation process, applying the classic pour-plate method. The samples taken from the mortar were suspended in a sterile 0.9% *w/v* NaCl solution at a ratio of 1:10. The suspensions obtained were used to prepare a set of serial dilutions. After preparing the dilution series, 0.1 mL of each dilution of 10^−1^ to 10^−4^ was spread on plates containing yeast mannitol agar (YMA, Sigma-Aldrich, St. Louis, MI, USA) and tryptic soy agar (TSA, BD, Becton, Dickinson and Company, New York, NY, USA) culture medium. All plates were placed in the incubator for 2–5 days at 30 °C, and the number of colonies appearing on the plates was counted. To isolate the rhizosphere bacteria, 1 g of soil from the plant roots was added to 250 mL bottles containing 50 mL of liquid TSB medium. After thorough mixing, the bottles were incubated at 30 °C for 2–5 days. After incubation, 1 mL of the suspension was pipetted into a test tube containing 9 mL of sterile physiological saline. A series of dilutions (up to 10^−4^) was prepared from the suspension, and each dilution was spread on plates containing yeast mannitol agar (YMA, Sigma-Aldrich) and tryptic soy agar (TSA, BD) culture medium. The plates were then incubated at 30 °C for 3 days. Based on phenotypic characteristics, including color, shape, and colony morphology, as well as Gram staining results, the bacterial isolates were classified and subsequently stored at 4 °C for further analysis. For long-term preservation, the isolates were maintained in nutrient broth supplemented with 20% glycerol and stored at −80 °C.

Bacterial identification was performed through 16S rRNA gene sequencing, as described by [73]. Total DNA from the bacteria was extracted using the Genome Mini AX Bacteria Kit (A&A Biotechnology, Gdańsk, Poland) after initial incubation in 50 mg/mL lysozyme (Sigma) for 1 h at 37 °C. Sequences encoding a small subunit of rRNA were amplified in the PCR reaction. PCR products were purified using the Clean-up Kit (A&A Biotechnology, Gdańsk, Poland) and sequenced at Genomed, Warsaw, Poland. The sequences obtained were then arranged into contigs and identified in BLAST + 2.15.0 services of the GenBank database. The selection of bacterial strains for plant studies was based on 16S rRNA identification and laboratory assessments of fungistatic activity against *Fusarium graminearum*, *Fusarium culmorum*, *Fusarium avenaceum*, and *Fusarium fujikuroi*. All strains of microorganisms used in the study were deposited at the Polish Collection of Microorganisms, located at the Institute of Immunology and Experimental Therapy in Wrocław. The collection is registered with the World Federation of Culture Collections (WFCC No. 106) and with the European Culture Collections Organization (ECCO).

### 4.2. Production and Standardization of Biopreparations

Bacterial cells were cultivated in a laboratory-scale bioreactor (Biostat Bplus, Sartorius AG, Göttingen, Germany) with a working volume of 5 L. The biosynthesis medium for biocontrol agents consisted of glucose (30 g/L), soy peptone (10 g/L), yeast extract (5 g/L), K_2_HPO_4_ (0.5 g/L), MgSO_4_·H_2_O (2 g/L), MgSO_4_·H_2_O (0.004 g/L), CaCl_2_ (0.005 g/L), and FeSO_4_·7H_2_O (0.0025 g/L). The pH of the medium was adjusted to 7.0 before sterilization, which was performed by autoclaving at 121 °C for 30 min. Inoculation was carried out under sterile conditions, with the inoculum volume set at 10% of the total medium volume. The bacterial cultures were incubated in the bioreactor at 30 °C for 72 h. During cultivation, the pH was automatically maintained at 7.0 using a dosing system that added either 0.1% HCl or 0.1% NaOH as needed. Oxygen saturation was maintained at 20% of dissolved oxygen by continuous aeration at a rate of 1 vessel volume per minute (vvm) and agitation speeds ranging from 200 to 1000 rpm. These optimal cultivation conditions were determined in preliminary experiments. After cultivation, samples were collected to quantify the number of vegetative cells and spores. The post-culture fluid was removed from the bioreactor and stored at 4 °C in a cold room until further processing. The bacterial cell count was determined using the pour-plate method with Tryptone Soy Agar (TSA, Becton, Dickinson and Co., Franklin Lakes, NJ, USA). Plates were incubated at 30 °C for 48 h, and the resulting colonies were counted to calculate the colony-forming units per milliliter (CFU/mL). Based on these findings, the preparations were standardized by diluting with sterile water to obtain a final cell concentration of 5 × 10^8^ CFU/mL for each bacterial strain. Mixtures of selected bacterial strains prepared using sterile saline were used to dilute and adjust the cell suspensions to the desired concentration. This approach resulted in a uniform initial bacterial load in all the treatments tested for single- and multi-strain inoculations, thus enabling a reliable comparative analysis.

### 4.3. Quantitative Assessment of Phosphate Solubilization

The ability of bacterial strains to solubilize phosphate was quantitatively evaluated using a standardized protocol. Initially, 48 h bacterial suspensions were centrifuged at 4500 rpm for 10 min to eliminate dissolved phosphates present in the culture medium, thereby preventing interference with the results. The supernatant was carefully decanted, and the remaining biomass was resuspended in 6 mL of sterile water. Before inoculation, the culture was standardized according to the following procedure: 6 mL of the inoculum culture was centrifuged, and the resulting biomass was resuspended in sterile distilled water to achieve an optical density corresponding to a cell concentration of 5 × 10^8^ CFU/mL (based on a calibration curve generated using a densitometer). A 2 mL aliquot of this standardized cell suspension was used as the inoculum for the PVK medium. For the experiment, 100 mL of liquid Pikovskaya (PVK) medium, supplemented with 2.5 g/L tricalcium phosphate (TCP) as an insoluble inorganic phosphorus source [74], was prepared. The medium was inoculated with 2 mL of the prepared bacterial suspension. The cultures were incubated at 30 °C under continuous shaking for 6 days to promote phosphate solubilization. Samples were aseptically collected at designated intervals and centrifuged at 4500 rpm for 15 min to remove sediment. The concentration of soluble phosphorus in the supernatant was quantified using the SPECTROQUANT Phosphate Cell Test (catalog no. 1.00673.0001; detection range: 9–307 mg/L PO_4_^3−^; Merck, Darmstadt, Germany). In each assay, 0.20 mL of the sample was transferred to a reaction vial containing one dose of P-1K reagent, mixed thoroughly, and heated at 120 °C for 30 min using a thermoreactor (Spectroquant TR 420; Merck, Darmstadt, Germany). After cooling to room temperature, 5 drops of P-2K reagent were added and mixed, followed by the addition of one dose of P-3K reagent. The mixture was allowed to stand for 5 min. Subsequently, the concentration of PO_4_^3−^ in the samples was measured using a spectrophotometer (Spectroquant Pharo 100; Merck, Darmstadt, Germany). This methodology provided precise and reproducible measurements of phosphate solubilization by the bacterial strains under investigation.

### 4.4. Evaluation of the Antifungal Activity of Bacterial Strains Against Phytopathogenic Fusarium Species

The antifungal potential of the bacterial strains was assessed against four major phytopathogenic fungi: *Fusarium culmorum*, *Fusarium graminearum*, *Fusarium fujikuroi*, and *Fusarium avenaceum*, which are significant contributors to crop yield losses. Bacterial cultures were grown for 48 h to reach a cell density of 1 × 10^8^ CFU/mL. Following cultivation, the bacterial suspension was divided into two fractions: one was used as-is, while the other was processed by centrifugation at 4500 rpm for 10 min. The supernatant from the centrifugation step was subsequently filtered through syringe filters with a pore size of 0.45 µm (Merck, Darmstadt, Germany). The antagonistic activity of the bacterial strains was evaluated using the well diffusion method. Petri dishes containing 20 mL of potato dextrose agar (PDA, Oxoid, Milan, Italy) were prepared at 55 °C and inoculated with 2% fungal inoculum. Once the medium had solidified, two wells, each with a diameter of 10 mm, were made in the agar. One well was filled with 100 µL of the 48 h bacterial culture, while the other was filled with 100 µL of the sterile, filtered supernatant. Plates were then incubated at 26 °C for 4 days. The inhibitory effects of the bacterial strains on fungal growth were determined by measuring the diameter of the inhibition zones surrounding the wells (in mm).

### 4.5. Seed Germination and Health Parameters of Plant Seedlings (Seed Inoculated with Fusarium)

The experiment consisted of determining the vigor index of winter wheat and maize that were inoculated with pathogens of the *Fusarium* genus and treated with various microbiological preparations. The rolled towel test, the method described in detail by Prasad [75], was performed in quadruple for each combination. One repetition contained 25 grains of winter wheat (*Tricticum aestivum* L.) of the Banatus variety and maize (*Zea mays* L.) of the Farmurmel variety. In this part of the experiment, plant material inoculated with pathogens was used. The grain was first inoculated with a suspension of mycelium and spores of fungi of the *Fusarium* genus at a spore concentration of 10^6^ in 1 mL. The inoculum consisted of fungal isolates obtained as a result of culturing pure cultures from wheat crops: *Fusarium avenaceum*, *Fusarium culmorum*, *Fusarium graminearum*, and *Fusarium fujikuroi*. Pathogen isolates with the highest plant infection rate were selected for the study and then multiplied on appropriate media. The pathogens used to inoculate the grain came from the collections of the Department of Microbiology of the Institute of Plant Protection—National Research Institute (IPP—NRI) in Poznan. In the other combinations, various strains of selected bacteria and a synthetic fungicide were applied to infected winter wheat grain. Based on 100 kg of grain, the following were used: *Bacillus velezensis_KT27* in doses of 0.5 and 1 L; *Paenibacillus polymyxa* in doses of 0.5 and 1 L; *Pseudomonas synxatha* in doses of 0.5 and 1 L; a mixture containing *Bacillus subtilis*, *Pseudomonas simiae*, and *Bacillus velezensis_S103* in doses of 0.5 and 1 L; and protioconazole (Gamelan 100 FS, Innvigo Sp. z o. o., protioconazole-100 g × L^−1^) in a dose of 100 mL (Table 6). The grain was subjected to laboratory dressing in a HEGE 11 liquid seed treater (Wintersteiger, Ried im Innkreis, Austria). The control sample was not treated. Individual combinations in the form of filter paper rolls were placed in a thermostatic cabinet with constant humidity and temperature parameters. The temperature inside the chamber was 21 °C. Four days after the experiment had been set up, the germination energy of winter wheat and maize grains was assessed. Germination capacity is the amount of a plant’s seeds that will normally germinate under optimal conditions for a given species. Germination energy is the number of seeds that germinate quickly. The germination capacity and germination energy are expressed as percentages [76].

The germination capacity of grains, and the length of shoots and roots of the tested plant material were assessed after 7 days. The obtained results were used to determine the vigor index. The vigor index (VI) was analyzed using the Abdul Baki and Anderson method [77].

Vigor index (VI) = [seeding length (cm) × germination (%)]

Infection of seedlings with pathogenic fungi was also assessed visually, and the percentage and infection rate were determined.

Infection index = (n(II) × 0.25) + n(III) × 0.75 + n(IV)n(I + II + III + IV)

where

I. no symptoms;
II. less than 50% of seedling attacked;
III. more than 50% seedling attacked;
IV. 100% of seedling attacked.

### 4.6. Effect of Selected Bacterial Strains on Plant Growth Under Greenhouse Conditions

The winter wheat (*Tricticum aestivum* L.) cv. Banatus and maize (*Zea mays* L.) cv. Farmurmel were grown for 31 days in a greenhouse belonging to the Department of Agronomy, the Poznań University of Life Sciences (52.482854, 16.900465). The conditions prevailing throughout the entire experiment were as follows: a photoperiod of 16 h of daylight and 8 h of darkness. The temperature was 25 ± 2 °C during the day and 20 ± 2 °C at night. LED lamps (EKO-LED Brzeziński, Kozicki Sp. k.—SOWELO, Józefosław, Poland) were used to supplement natural light. The water in the soil was replenished using tap water, and the water capacity was maintained at 65–75%. Humidity was 50–80%.

Healthy winter wheat and maize seeds were treated with various strains of selected bacteria and a synthetic agent. The control sample was not treated. Based on 100 kg of grain, the following were used: *Bacillus velezensis_KT27* in doses of 0.5 and 1 L; *Paenibacillus polymyxa* in doses of 0.5 and 1 L; *Pseudomonas synxatha* in doses of 0.5 and 1 L; a mixture containing *Bacillus subtilis*, *Pseudomonas simiae*, and *Bacillus velezensis_S103* in doses of 0.5 and 1 L; protioconazole (Gamelan 100 FS, Innvigo Sp. z o. o., protioconazole- 100 g × L^−1^) in a dose of 100 mL (Table 6). The treated seeds were sown to a depth of 2 cm in 4 replications (10 seeds for each replication) for winter wheat, and to a depth of 5 cm in 4 replications (4 seeds for each replication) for maize, for each combination. The seeds were sown into production pots with a capacity of 1 L and a diameter of 15 cm. The substrate was fine-structured soil from frozen peat from Lasland sp. z o. o (Grady, Poland).

Plant chlorophyll fluorescence was measured 21 days after sowing. A Multi-Mode Chlorophyll Fluorometer OS5p (Optisciences Inc., Hudson, NH, USA) with the selected protocol was used to perform this measurement. Before measurement, the modulation intensity parameter and the detector gain value were set so that the fluorescence signal was in the range of 150–250 counts and was stable. This signal value means that the lighting does not affect the photosynthesis process in any way. After 30 min of adaptation in the dark, the following parameters were measured: Fv—variable fluorescence, Fm—maximum fluorescence, and Fv/Fm—maximum photochemical efficiency of PSII. Fluorescence was measured on the youngest fully developed leaf of the plant with three replicates/pot for wheat and two replicates/pot for maize for each combination in one measurement series.

Height measurements (cm plant^−1^) of winter wheat and maize plants were performed 18 and 28 days after sowing. Eight plants in four replications for winter wheat and three plants in four replications for maize for each combination were measured. The last measurement determined the fresh weight of plants. The plants were cut at the level of the soil line and weighed using a laboratory scale (RADWAG Scales, Radom, Poland). Eight plants were weighed from each pot for winter wheat and three plants for maize, respectively.

The average results of the tested combinations were subjected to statistical analysis using the Statistica program (version 12, StatSoft Inc., Tulsa, OK, USA). For the obtained results, ANOVA analysis and Tukey’s test with protected LSD were performed at a probability level of 0.05.

## 5. Conclusions

Research on biological solutions applied to protect and stimulate plant growth is important for environmental reasons, in view of the withdrawal of various active substances and the problems of pathogen resistance. The obtained results indicate that individual preparations containing *Bacillus* spp., *Paenibacillus* spp., and *Pseudomonas* spp. are effective against *Fusarium* pathogens, although statistically less effective than synthetic triazole. Moreover, the study provided evidence of a positive correlation between various growth parameters of crop plants inoculated with selected bacteria *Bacillus velezensis_KT27*, *Paenibacillus polymyxa*, *Pseudomonas synxatha*, and a mixture *of Bacillus subtilis*, *Pseudomonas simiae*, and *Bacillus velezensis_S103.* The level of efficacy in stimulating the growth of winter wheat and maize depended on the type of crop, dose, and type of microorganisms belonging to the plant-growth-promoting rhizobacteria group. These microorganisms can provide an alternative solution to improving plant health and growth while maintaining a safe environment. The obtained results contribute to increasing knowledge concerning the efficacy of selected bacteria with fungistatic and biostimulatory properties. Such knowledge can be an inspiration for other scientists and contribute to the development of research on local bacterial communities. Based on the presented data, it is recommended to conduct additional research under uncontrolled field conditions.

## Figures and Tables

**Table 1:** Evaluation of the antifungal activity of bacterial strains against phytopathogenic *Fusarium* species.

Fraction	Bacterial Strain	The Diameter of the Clear Zone [mm]			
		*Fusarium culmorum*	*Fusarium graminearum*	*Fusarium fujikuori*	*Fusarium avenaceum*
Culture fluid containing bacterial cells	*Bacillus velenensis_KT27*	0	50	26	14
	*Paenibacillus polymyxa*	0	50	50	14
	*Pseudomonas synxantha*	0	0	16	20
	*Bacillus subtilis*	12	0	24	14
	*Bacillus velenensis_S103*	0	0	22	14
	*Pseudomonas simiae*	12	0	18	0
Cell-free supernatant post-centrifugation and post-filtration	*Bacillus velenensis*	0	50	24	12
	*Paenibacillus polymyxa*	0	40	40	12
	*Pseudomonas synxantha*	0	0	14	16
	*Bacillus subtilis*	0	0	20	12
	*Bacillus velenensis_S103*	0	0	18	12
	*Pseudomonas simiae*	0	0	16	0

**Table 2:** The effect of Bacillus ssp., Paenibacillus ssp., Pseudomonas ssp., or the synthetic fungicide on the germination energy and germination capacity of winter wheat and maize grain inoculated with Fusarium.

No.	Preparation	Dose per 100 kg of Grain (L)	Winter Wheat		Maize	
			Germination Energy (%)	Germination Capacity (%)	Germination Energy (%)	Germination Capacity (%)
1	Control	-	96 a	98 a	95 a	95 a
2	Prothioconazole	0.1	96 a	96 a	95 a	96 a
3	*Bacillus velezensis_KT27*	0.5	96 a	96 a	95 a	96 a
4	*Bacillus velezensis_KT27*	1.0	96 a	97 a	92 a	95 a
5	*Paenibacillus polymyxa*	0.5	94 ab	97 a	95 a	96 a
6	*Paenibacillus polymyxa*	1.0	93 ab	95 a	94 a	95 a
7	*Pseudomonas synxatha*	0.5	93 ab	96 a	97 a	98 a
8	*Pseudomonas synxatha*	1.0	95 ab	97 a	93 a	95 a
9	*Bacillus subtilis*, *Pseudomonas simiae*, *Bacillus velezensis_S103*	0.5	97 a	97 a	96 a	96 a
10	*Bacillus subtilis*, *Pseudomonas simiae*, *Bacillus velezensis_S103*	1.0	90.5 b	94 a	94 a	94 a

Different letters indicate statistically significant differences in mean values based on the LSD test (*p* < 0.05).

**Table 3:** The effect of *Bacillus* ssp., *Paenibacillus* ssp., *Pseudomonas* ssp., or the synthetic fungicide on the height of winter wheat and maize plants under greenhouse conditions.

No.	Preparation	Dose per 100 kg of Grain (L)	Winter Wheat Height (cm)		Maize Height (cm)	
			18 DAS *	28 DAS *	18 DAS *	28 DAS *
1	Control	-	10.85 bcd	30.56 ab	20.69 d	70.46 c
2	Prothioconazole	0.1	10.50 d	30.65 ab	21.35 cd	70.71 bc
3	*Bacillus velezensis_KT27*	0.5	11.38 abc	31.39 a	23.26 b	73.61 a
4	*Bacillus velezensis_KT27*	1.0	11.52 ab	31.05 ab	22.46 b	72.28 abc
5	*Paenibacillus polymyxa*	0.5	11.55 a	30.70 ab	23.02 b	73.42 ab
6	*Paenibacillus polymyxa*	1.0	10.90 a–d	30.26 b	23.03 b	73.53 ab
7	*Pseudomonas synxatha*	0.5	10.98 a–d	29.39 c	24.28 a	74.96 a
8	*Pseudomonas synxatha*	1.0	11.56 a	30.45 b	22.70 b	72.80 abc
9	*Bacillus subtilis*, *Pseudomonas simiae*, *Bacillus velezensis_S103*	0.5	10.82 cd	30.63 ab	22.28 bc	72.68 abc
10	*Bacillus subtilis*, *Pseudomonas simiae*, *Bacillus velezensis_S103*	1.0	11.25 abc	30.49 b	22.52 b	72.60 abc

* Days after sowing. Different letters indicate statistically significant differences in mean values based on the LSD test (*p* < 0.05).

**Table 4:** The effect of *Bacillus* ssp., *Paenibacillus* ssp., *Pseudomonas* ssp., or the synthetic fungicide on Fv/Fm—maximum photochemical efficiency of photosystem II (non-nominated units) in winter wheat and maize plants.

No.	Preparation	Dose per 100 kg of Grain (L)	Winter Wheat	Maize
			Fv/Fm	Fv/Fm
1	Control	-	0.808 d	0.787 a
2	Prothioconazole	0.1	0.810 cd	0.784 a
3	*Bacillus velezensis_KT27*	0.5	0.810 cd	0.789 a
4	*Bacillus velezensis_KT27*	1.0	0.813 abc	0.787 a
5	*Paenibacillus polymyxa*	0.5	0.811 bcd	0.791 a
6	*Paenibacillus polymyxa*	1.0	0.813 abc	0.793 a
7	*Pseudomonas synxatha*	0.5	0.816 a	0.790 a
8	*Pseudomonas synxatha*	1.0	0.814 ab	0.793 a
9	*Bacillus subtilis*, *Pseudomonas simiae*, *Bacillus velezensis_S103*	0.5	0.815 a	0.792 a
10	*Bacillus subtilis*, *Pseudomonas simiae*, *Bacillus velezensis_S103*	1.0	0.812 a–d	0.788 a

Different letters indicate statistically significant differences in mean values based on the LSD test (*p* < 0.05).

**Table 5:** The effect of *Bacillus* ssp., *Paenibacillus* ssp., *Pseudomonas* ssp., or the synthetic fungicide on the fresh weight of winter wheat and maize plants.

No.	Preparation	Dose per 100 kg of Grain (L)	Winter Wheat	Maize
			Plant Fresh Weight (g)	Plant Fresh Weight (g)
1	Control	-	8.29 d	40.18 cd
2	Prothioconazole	0.1	8.75 cd	40.10 d
3	*Bacillus velezensis_KT27*	0.5	9.81 ab	45.40 ab
4	*Bacillus velezensis_KT27*	1.0	10.12 a	44.65 ab
5	*Paenibacillus polymyxa*	0.5	9.61 abc	47.20 ab
6	*Paenibacillus polymyxa*	1.0	10.02 ab	45.05 ab
7	*Pseudomonas synxatha*	0.5	9.07 bcd	47.47 a
8	*Pseudomonas synxatha*	1.0	9.26 a–d	44.96 ab
9	*Bacillus subtilis*, *Pseudomonas simiae*, *Bacillus velezensis_S103*	0.5	8.81 cd	44.55 ab
10	*Bacillus subtilis*, *Pseudomonas simiae*, *Bacillus velezensis_S103*	1.0	8.42 d	43.66 bc

Different letters indicate statistically significant differences in mean values based on the LSD test (*p* < 0.05).

**Table 6:** Preparations used, containing *Bacillus* spp., *Paenibacillus* spp., *Pseudomonas* spp., or synthetic fungicide, and their dosages (L) × 100 kg^−1^ grain.

No.	Preparation	Dose per 100 kg of Grain (L)
1	Control	-
2	Prothioconazole	0.1
3	*Bacillus velezensis_KT27*	0.5
4	*Bacillus velezensis_KT27*	1.0
5	*Paenibacillus polymyxa*	0.5
6	*Paenibacillus polymyxa*	1.0
7	*Pseudomonas synxatha*	0.5
8	*Pseudomonas synxatha*	1.0
9	*Bacillus subtilis*, *Pseudomonas simiae*, *Bacillus velezensis_S103*	0.5
10	*Bacillus subtilis*, *Pseudomonas simiae*, *Bacillus velezensis_S103*	1.0

## Data Availability

The source data is stored by the authors and will be available for readers if necessary.

## References

[1] Hatfield J.L., Dold C. (2018). Agroclimatology and Wheat Production: Coping with Climate Change. Front. Plant Sci..

[2] Podgórska-Kryszczuk I., Solarska E., Kordowska-Wiater M. (2022). Biological Control of *Fusarium culmorum, Fusarium graminearum* and *Fusarium poae* by Antagonistic Yeasts. Pathogens.

[3] Jakhar D.S., Singh R. (2015). Biotic stress response in maize (*Zea mays* L.). J. Biotechnol. Crop Sci..

[4] Baillo E.H., Kimotho R.N., Zhang Z., Xu P. (2019). Transcription Factors Associated with Abiotic and Biotic Stress Tolerance and Their Potential for Crops Improvement. Genes.

[5] Suzuki N., Rivero R.M., Shulaev V., Blumwald E., Mittler R. (2014). Abiotic and biotic stress combinations. New Phytol..

[6] Shuping D.S.S., Eloff J.N. (2017). The use of plants to protect plants and food against fungal pathogenes: A review. Afr. J. Tradit. Complement. Altern. Med..

[7] Martinelli F., Scalenghe R., Davino S., Panno S., Scuderi G., Ruisi P., Villa P., Stroppiana D., Boschetti M., Goulart L.R. (2015). Advanced methods of plant disease detection. A review. Agron. Sustain. Dev..

[8] Karlsson I., Persson P., Friberg H. (2021). *Fusarium* Head Blight from a Microbiome Perspective. Front. Microbiol..

[9] Chandler E.A., Simpson D.R., Thomsett M.A., Nicholson P. (2003). Development of PCR assays to *Tri7* and *Tri13* trichothecene biosynthetic genes, and characterisation of chemotypes of *Fusarium graminearum*, *Fusarium culmorum* and *Fusarium cerealis*. Physiol. Mol. Plant Pathol..

[10] Kosiak B., Torp M., Skjerve E., Thrane U. (2003). The Prevalence and Distribution of *Fusarium* species in Norwegian Cereals: A Survey. Acta Agric. Scand..

[11] SØrensen J.N., Phipps R.K., Nielsen K.F., Schroers H.J., Frank J., Thrane U. (2009). Analysis of *Fusarium avenaceum* Metabolites Produced during Wet Apple Core Rot. J. Agric. Food Chem..

[12] Janevska S., Tudzynski B. (2018). Secondary metabolism in *Fusarium fujikuroi*: Strategies to unravel the function of biosynthetic pathways. Appl. Microbiol. Biotechnol..

[13] Kardava K., Tetz V., Vecherkovskaya M., Tetz G. (2023). Seed dressing with M451 promotes seedling growth in wheat and reduces root phytopathogenic fungi without affecting endophytes. Front. Plant Sci..

[14] Tonin R.B., Reis E.M., Avozani A. (2017). Reduction in the in vitro sensitivity oF Drechslera tritici-repentis, isolated from wheat, to strobilurin and triazole fungicides. Summa Phytopathol..

[15] Odds F.C., Brown A.J.P., Gow N.A.R. (2003). Antifungal agents: Mechanisms of action. Trends Microbiol..

[16] Alcazar-Fuoli L., Mellado E. (2013). Ergosterol biosynthesis in *Aspergillus fumigatus*: Its relevance as an antifungal target and role in antifungal drug resistance. Front. Microbiol..

[17] Garcia-Ruiz H., Szurek B., Van den Ackerveken G. (2021). Stop helping pathogens: Engineering plant susceptibility genes for durable resistance. Curr. Opin. Biotechnol..

[18] Bainsla N.K., Meena H.P. (2016). Breeding for Resistance to Biotic Stresses in Plants. J. Plant Stress Physiol..

[19] Lahlali R., Ezrari S., Radouane N., Kenfaoui J., Esmaeel Q., El Hamss H., Belabess Z., Barka E.A. (2022). Biological Control of Plant Pathogens: A Global Perspective. Microorganisms.

[20] Lengai G.M.W., Muthomi J.W. (2018). Biopesticides and Their Role in Sustainable Agricultural Production. J. Biosci. Med..

[21] Kumar J., Ramlal A., Mallick D., Mishra V. (2021). An Overview of Some Biopesticides and Their Importance in Plant Protection for Commercial Acceptance. Plants.

[22] He D.-C., He M.-H., Amalin D.M., Liu W., Alvindia D.G., Zhan J. (2021). Biological Control of Plant Diseases: An Evolutionary and Eco-Economic Consideration. Pathogens.

[23] Rabbee M.F., Ali M.S., Choi J., Hwang B.S., Jeong S.C., Baek K.-H. (2019). *Bacillus velezensis:* A Valuable Member of Bioactive Molecules within Plant Microbiomes. Molecules.

[24] Timmusk S., Grantcharova N., Wagner E.G.H. (2005). *Paenibacillus polymyxa* Invades Plant Roots and Forms Biofilms. Appl. Environ. Microbiol..

[25] Daud N.S., Din A.R.J.M., Rosli M.A., Azam Z.M., Othman N.Z., Sarmidi M.R. (2019). Paenibacillus polymyxa bioactive compounds for agricultural and biotechnological applications. Biocatal. Agric. Biotechnol..

[26] Janakiev T., Dimkić I., Unković N., Ljaljević Grbić M., Opsenica D., Gašić U., Stanković S., Berić T. (2019). Phyllosphere Fungal Communities of Plum and Antifungal Activity of Indigenous Phenazine-Producing *Pseudomonas synxantha* Against *Monilinia laxa*. Front. Microbiol..

[27] Nithyapriya S., Lalitha S., Sayyed R.Z., Reddy M.S., Dailin D.J., El Enshasy H.A., Luh Suriani N., Herlambang S. (2021). Production, Purification, and Characterization of Bacillibactin Siderophore of *Bacillus subtilis* and Its Application for Improvement in Plant Growth and Oil Content in Sesame. Sustainability.

[28] Pieterse C.M.J., Berendsen R.L., de Jonge R., Stringlis I.A., Van Dijken A.J.H., Van Pelt J.A., Van Wees S.C.M., Yu K., Zamioudis C., Bakker P.A.H.M. (2021). *Pseudomonas simiae* WCS417: Star track of a model beneficial rhizobacterium. Plant Soil.

[29] Cetner M.D., Dąbrowski P., Samborska I.A., Łukasik I., Swoczyna T., Pietkiewicz S., Bąba W., Kalaji H.M. (2016). Application of chlorophyll fluorescence measurements in environmental research. Kosm. Probl. Nauk. Biol..

[30] Murchie E.H., Lawson T. (2013). Chlorophyll fluorescence analysis: A guide to good practice and understanding some new applications. J. Exp. Bot..

[31] Sobiech Ł., Grzanka M., Kurasiak-Popowska D., Radzikowska D. (2020). Phytotoxic Effect of Herbicides on Various Camelina [*Camelina sativa* (L.) Crantz] Genotypes and Plant Chlorophyll Fluorescence. Agriculture.

[32] Huang S., Luo H., Ashraf U., Abrar M., He L., Zheng A., Wang Z., Zhang T., Tang X. (2019). Seed Treatment with Paclobutrazol Affects Early Growth, Photosynthesis, Chlorophyll Fluorescence and Physiology of Rice. Appl. Ecol. Environ. Res..

[33] Maxwell K., Johnson G.N. (2000). Chlorophyll fluorescence—A practical guide. J. Exp. Bot..

[34] Khaeim H.M., Clark A., Pearson T., Van Sanford D. (2019). Methods of Assessing *Fusarium* Damage to Wheat Kernels. J. For. Agric. Sci..

[35] Moumni M., Brodal G., Romanazzi G. (2023). Recent innovative seed treatment methods in the management of seedborne pathogens. Food Secur..

[36] Ismail M.A., Amin M.A., Eid A.M., Hassan S.E.D., Mahgoub H.A., Lashin I., Abdelwahab A.T., Azab E., Gobouri A.A., Elkelish A. (2021). Comparative Study between Exogenously Applied Plant Growth Hormones versus Metabolites of Microbial Endophytes as Plant Growth-Promoting for *Phaseolus vulgaris* L.. Cells.

[37] Chen L., Wu Y.D., Chong X.Y., Xin Q.H., Wang D.X., Bian K. (2020). Seed-borne endophytic *Bacillus velezensis* LHSB1 mediate the biocontrol of peanut stem rot caused by *Sclerotium rolfsii*. J. Appl. Microbiol..

[38] Khan N., Ali S., Shahid M.A., Mustafa A., Sayyed R.Z., Curá J.A. (2021). Insights into the Interactions among Roots, Rhizosphere, and Rhizobacteria for Improving Plant Growth and Tolerance to Abiotic Stresses: A Review. Cells..

[39] Borah A., Thakur D. (2020). Phylogenetic and Functional Characterization of Culturable Endophytic Actinobacteria Associated with *Camellia* spp. for Growth Promotion in Commercial Tea Cultivars. Front. Microbiol..

[40] Crowther T.W., van den Hoogen J., Wan J., Mayes M.A., Keiser A.D., Mo I., Averill C., Maynard D.S. (2019). The global soil community and its influence on biogeochemistry. Science.

[41] Phi Q.-T., Park Y.-M., Seul K.-J., Ryu C.-M., Park S.-H., Kim J.-G., Ghim S.-Y. (2010). Assessment of rootassociated *Paenibacillus polymyxa* groups on growth promotion and induced systemic resistance in pepper. J. Microbiol. Biotechnol..

[42] Yerkovich N., Cantoro R., Palazzini J.M., Torres A., Chulze S.N. (2020). *Fusarium* head blight in Argentina: Pathogen aggressiveness, triazole tolerance and biocontrol-cultivar combined strategy to reduce disease and deoxynivalenol in wheat. Crop Prot..

[43] Avozani A., Tonin R.B., Reis E.M., Camera J., Ranzi C. (2014). In vitro sensitivity of *Fusarium graminearum* isolates to fungicides. Summa Phytopathol..

[44] Sooväli P., Koppel M., Kangor T. (2017). Effectiveness of seed treatment against *Fusarium* spp. and *Cochliobolus sativus* of spring barley in different conditions. Agron. Res..

[45] Lucas J.A., Hawkins N.J., Fraaije B.A. (2015). The evolution of fungicide resistance. Adv. Appl. Microbiol..

[46] Carvalho F.P. (2017). Pesticides, environment, and food safety. Food Energy Secur..

[47] Horikoshi K., Grant W.D. (1998). Extremophiles: Microbial Life in Extreme Environments.

[48] Sagova-Mareckova M., Boenigk J., Bouchez A., Cermakova K., Chonova T., Cordier T., Eisendle U., Elersek T., Fazi S., Fleituch T. (2021). Expanding Ecological Assessment by Integrating Microorganisms into Routine Freshwater Biomonitoring. Water Res..

[49] Chen L., Heng J., Qin S., Bian K. (2018). A comprehensive understanding of the biocontrol potential of *Bacillus velezensis* LM2303 against *Fusarium* head blight. PLoS ONE.

[50] Agha S.I., Jahan N., Azeem S., Parveen S., Tabassum B., Raheem A., Ullah H., Khan A. (2022). Research article Characterization of broad-spectrum biocontrol efficacy of *Bacillus velezensis* against *Fusarium oxysporum* in *Triticum aestivum* L.. Not. Bot. Horti Agrobot. Cluj-Napoca.

[51] Timmusk S., Copolovici D., Copolovici L., Teder T., Nevo E., Behers L. (2019). *Paenibacillus polymyxa* bioflm polysaccharides antagonise *Fusarium graminearum*. Sci. Rep..

[52] Baard V., Bakare O.O., Daniel A.I., Nkomo M., Gokul A., Keyster M., Klein A. (2023). Biocontrol Potential of *Bacillus subtilis* and *Bacillus tequilensis* against Four *Fusarium* Species. Pathogens.

[53] Wang J., Liu J., Chen H., Yao J. (2007). Characterization of *Fusarium graminearum* inhibitory lipopeptide from *Bacillus subtilis* IB. Appl. Microbiol. Biotechnol..

[54] Gu Q., Yang Y., Yuan Q., Shi G., Wu L., Lou Z., Huo R., Wu H., Borriss R., Gao X. (2017). Bacillomycin D produced by *Bacillus amyloliquefaciens* is involved in the antagonistic interaction with the plantpathogenic fungus *Fusarium graminearum*. Appl. Environ. Microbiol..

[55] Gong A.-D., Li H.-P., Yuan Q.-S., Song X.-S., Yao W., He W.-J., Zhang J.-B., Liao Y.-C. (2015). Antagonistic Mechanism of Iturin A and Plipastatin A from *Bacillus amyloliquefaciens* S76-3 from Wheat Spikes against *Fusarium graminearum*. PLoS ONE.

[56] Sarić-Krsmanović M.M., Bozic D.M., Radivojevic L.M., Gajić Umiljendić J.S., Santric L., Vrbničanin S.P. (2017). Effects of plant growth promoting rhizobacteria (PGPR) and cover crops on seed germination and early establishment of field dodder (*Cuscuta campestris* Yunk.) and early establishment of field dodder (*Cuscuta campestris* Yunk.). Pestic. Fitomed..

[57] Jha K.S., Saraf M. (2011). Effect of Plant Growth Promoting Rhizobacteria on Seed Germination Behaviour and Seedling Vigor of Jatropha Curcas. Int. J. Biotechnol. Biosci..

[58] Meng Q., Jiang H., Hao J.J. (2016). Effects of *Bacillus velezensis* strain BAC03 in promoting plant growth. Biol. Control.

[59] Wang J., Qu F., Liang J., Yang M., Hu X. (2022). *Bacillus velezensis* SX13 promoted cucumber growth and production by accelerating the absorption of nutrients and increasing plant photosynthetic metabolism. Sci. Hortic..

[60] Mosela M., Andrade G., Massucato L.R., Almeida S.R.d.A., Nogueira A.F., Filho R.B.d.L., Zeffa D.M., Mian S., Higashi A.Y., Shimizu G.D. (2022). *Bacillus velezensis* strain Ag75 as a new multifunctional agent for biocontrol, phosphate solubilization and growth promotion in maize and soybean crops. Sci. Rep..

[61] Chaturvedi H., Singh B., Jajoo A., Prakash A. (2022). Shielding of Photosynthetic Apparatus by Consortia of Bacterial Endophytes in Tomato Plants Suffering From *Fusarium* Wilt. Front. Agron..

[62] Padda K.P., Puri A., Chanway C.P. (2016). Plant growth promotion and nitrogen fixation in canola by an endophytic strain of *Paenibacillus polymyxa* and its GFPtagged derivative in a long-term study. Botany.

[63] Minut M., Diaconu M., Rosca M., Cozma P., Bulgariu L., Gavrilescu M. (2023). Screening of *Azotobacter, Bacillus* and *Pseudomonas* Species as Plant Growth-Promoting Bacteria. Processes.

[64] Ganesh J., Hewitt K., Devkota A.R., Wilson T., Kaundal A. (2024). IAA-producing plant growth promoting rhizobacteria from *Ceanothus velutinus* enhance cutting propagation efficiency and Arabidopsis biomass. Front. Plant Sci..

[65] Li C., Zeng Q., Han Y., Zhou X., Xu H. (2024). Effects of *Bacillus subtilis* on Cucumber Seedling Growth and Photosynthetic System under Different Potassium Ion Levels. Biology.

[66] Wu B., Wang X., Yang L., Yang H., Zeng H., Qiu Y., Wang C., Yu J., Li J., Xu D. (2016). Effects of *Bacillus amyloliquefaciens* ZM9 on bacterial wilt and rhizosphere microbial communities of tobacco. Appl. Soil Ecol..

[67] Çelİkten M., Bozkurt İ.A. (2018). Determination of efficacies of bacteria isolated from wheat rhizospheres on plant growth. Ziraat Fakültesi Derg..

[68] Schmidt C.S., Mrnka L., Frantík T., Lovecká P., Vosátka M. (2018). Plant growth promotion of *Miscanthus*  ×  *giganteus* by endophytic bacteria and fungi on non-polluted and polluted soils. World J. Microbiol. Biotechnol..

[69] Hawkesford M.J., Cakmak I., Coskun D., De Kok L.J., Lambers H., Schjoerring J.K., White P.J. (2023). Functions of macronutrients. Marschner’s Mineral Nutrition of Plants.

[70] Che S., Xu Y., Qin X., Tian S., Wang J., Zhou X., Cao Z., Wang D., Wu M., Wu Z. (2024). Building microbial consortia to enhance straw degradation, phosphorus solubilization, and soil fertility for rice growth. Microb. Cell Factories.

[71] Luo D., Shi J., Li M., Chen J., Wang T., Zhang Q., Yang L., Zhu N., Wang Y. (2024). Consortium of Phosphorus-Solubilizing Bacteria Promotes Maize Growth and Changes the Microbial Community Composition of Rhizosphere Soil. Agronomy.

[72] Biostimulants Market Size, Share & Trends Analysis Report By Active Ingredients (Acid Based, Microbial), by Crop Type, by Application (Foliar, Soil Treatment); by Region, and Segment Forecasts, 2023–2030; Report ID: GVR-2-68038-346-1; San Francisco, CA, USA. https://www.grandviewresearch.com/industry-analysis/biostimulants-market.

[73] Wita A., Białas W., Wilk R., Szychowska K., Czaczyk K. (2019). The Influence of Temperature and Nitrogen Source on Cellulolytic Potential of Microbiota Isolated from Natural Environment. Pol. J. Microbiol..

[74] Pikovskaya R.I. (1948). Mobilization of Phosphorus in Soil Connection with the Vital Activity of Some Microbial Species. Microbiology.

[75] Prasad S.R., Dadlani D., Yadava K. (2023). Testing seed for quality. Seed Science and Technology.

[76] ISTA (2006). ISTA Handbook on Seedling Evaluation.

[77] Abdul-Baki A.A., Anderson J.D. (1973). Vigor determination in soybean seed by multiple criteria. Crop Sci..

